# Prognostic Value of ^18^F-Choline PET/CT in Patients with Metastatic Castration-Resistant Prostate Cancer Treated with Radium-223

**DOI:** 10.3390/biomedicines8120555

**Published:** 2020-11-30

**Authors:** Luca Filippi, Gian Paolo Spinelli, Agostino Chiaravalloti, Orazio Schillaci, Francesco Equitani, Oreste Bagni

**Affiliations:** 1 Department of Nuclear Medicine, “Santa Maria Goretti” Hospital, 04100 Latina, Italy; o.bagni@ausl.latina.it; 2 Oncology Unit, AUSL Latina (District 1) Sapienza University of Rome, 04011 Aprilia, Italy; gp.spinelli@ausl.latina.it; 3 Department of Biomedicine and Prevention, University Tor Vergata, 00133 Rome, Italy; agostino.chiaravalloti@uniroma2.it (A.C.); orazio.schillaci@uniroma2.it (O.S.); 4 IRCCS Neuromed, 86077 Pozzilli, Italy; 5 Department of Transfusion Medicine, Santa Maria Goretti Hospital, 04100 Latina, Italy; f.equitani@ausl.latina.it

**Keywords:** castration-resistant prostate cancer, molecular imaging, positron emission computed tomography, ^18^F-choline, theranostics, radium-223, bone metastases

## Abstract

We aimed to investigate the role of positron emission computed tomography (PET/CT) with ^18^F-choline for predicting the outcome of metastatic castration-resistant prostate cancer (mCRPC) submitted to treatment with Radium-223 (^223^Ra-therapy). Clinical records of 20 mCRPC patients submitted to PET/CT with ^18^F-choline before ^223^Ra-therapy were retrospectively evaluated. The following PET-derived parameters were calculated: number of lesions, maximum and mean standardized uptake values (SUVmax, SUVmean), lean body mass corrected SUV peak (SULpeak), metabolic tumor volume (MATV), and total lesion activity (TLA). After ^223^Ra-therapy, all patients underwent regular follow-up until death. The predictive power of clinical and PET-derived parameters on overall survival (OS) was assessed by Kaplan–Meier analysis and the Cox proportional hazard method. All the patients showed ^18^F-choline-avid lesions at baseline PET/CT. Among the enrolled subjects, eleven (55%) completed all the six scheduled cycles of ^223^Ra-therapy; seven (35%) were responders according to imaging and biochemical parameters. Mean OS was 12.7 ± 1.4 months: by Kaplan–Meier analysis, number of lesions, PSA level and TLA were significantly correlated with OS. In multivariate Cox analysis, TLA remained the only significant predictor of survival (*p* = 0.003; hazard ratio = 7.6, 95% confidence interval = 1.9–29.5 months). ^18^F-choline PET may be useful for patients’ stratification before ^223^Ra-therapy. In particular, high metabolically active tumor burden (i.e., TLA) was predictive of poor outcome.

## 1. Introduction

Prostate cancer is a leading cause of death in developed countries. In particular, the condition of hormone-refractory metastatic disease, termed as “castration-resistant prostate cancer” (CRPC), is characterized by poor prognosis, with a reported median overall survival after CRPC onset ranging 9–30 months among patients without metastases and 9–13 months among patients with metastases (mCRPC) [1]. In 2004, the US Food and Drug Administration (FDA) approved docetaxel as a front line treatment for mCRPC [2]. Since 2010, carbazitaxel has been further introduced to manage mCRPC patients progressing during docetaxel [3]. Taxane-based chemotherapy has represented the treatment of choice in mCRPC for many years and proved useful for improving survival and achieving pain control [4].

The therapeutic landscape of mCRPC has been enormously changed by the recent introduction of novel therapeutic tools, such as new-generation antiandrogen drugs, (i.e., abiraterone acetate and enzalutamide) [5]. Recently, other treatments have been implemented such as poly(ADP-ribose) polymerase inhibitors (e.g., olaparib), which were demonstrated to provide survival benefit in mCRPC patients bearing defects in DNA repair genes, and immunotherapy with sipuleucel-T, consisting of autologous peripheral blood mononuclear cells, pulsed ex vivo and incubated with a fusion protein (prostatic acid phosphatase) [6,7]. Furthermore, prostate-specific membrane antigen (PSMA) has emerged as an effective molecular target for prostate cancer imaging and therapy, according to the so-called “theranostic” approach. In this regard, the radioligand PSMA-617 has been conjugated with the beta-emitting radioisotope lutetium-177 (^177^Lu) for the radionuclide therapy of mCRPC with favorable response rates in the first clinical trials [8].

Targeted alpha therapy (TAT) with the bone-seeking radiopharmaceutical 223radium-dichloride (^223^Ra-therapy) has been approved by the FDA and European Medical Agency (EMA) after the phase III clinical trial ALYSMPCA that demonstrated survival benefit and delayed the first symptomatic skeletal event in mCRPC patients with bone metastases submitted to ^223^Ra-therapy as compared to those undergoing standard of care treatment [9]. It has been reported that the outcome of ^223^Ra-therapy in clinical practice is strongly influenced by pre-treatment patients’ selection and prognostic stratification [10].

Several clinical parameters have been investigated as predictive biomarkers of outcome after ^223^Ra-therapy such as prostate specific antigen (PSA), hemoglobin level, Eastern Cooperative Oncology Group performance status (ECOG), neutrophil to lymphocyte ratio (NLR), alkaline phosphatase (ALP) and lactate dehydrogenase (LDH) concentration [11,12].

Positron emission computed tomography (PET/CT) with ^18^F-choline is a well-established imaging approach for the detection of prostate cancer recurrence after surgery/radiotherapy and has also been applied for the prognostication of mCRPC patients submitted to treatment with the novel antiandrogen drugs (i.e., enzalutamide and abiraterone acetate) [13]. Of note, PET-derived functional parameters, such as maximum and mean standardized uptake value (SUVmax and SUVmean) and lean body mass corrected SUV peak (SULpeak), have been investigated as potential biomarkers for patients’ prognostication before therapy. Furthermore, volumetric SUV-based parameters, namely Metabolically Active Tumor Volume (MATV) and Total Lesion Activity (TLA), both reflecting the burden of metabolically active disease, were found to have significant prognostic impact [13,14,15,16]. Nevertheless, the role of ^18^F-choline PET-derived biomarkers for patients’ selection before ^223^Ra-therapy has not been thoroughly investigated yet [14,15].

The aim of this retrospective, single-center, clinical study was to evaluate whether baseline PET/CT with ^18^F-choline, and in particular PET-derived parameters, may be applied to predict survival of mCRPC patients after ^223^Ra-therapy. Furthermore, as secondary endpoint of the study, we evaluated the role of ^18^F-choline PET/CT for the assessment of the response to treatment. 

## 2. Experimental Section

### 2.1. Study Design

Clinical records of patients affected by mCRPC, treated with ^223^Ra-therapy between January 2017 and September 2019 in our Nuclear Medicine Department, and submitted to PET/CT with ^18^F-choline at baseline and within 1 month after the end of treatment, were retrospectively reviewed. According to our standard procedure, all the patients signed a written informed consent form, which encompassed the use of anonymized data for retrospective research purposes before each PET/CT scan and before the start of ^223^Ra-therapy.

All the subjects had to fulfill the following inclusion criteria: (a) age over 18 years, (b) an initial histopathology diagnosis of prostate cancer, (c) fulfillment of clinical criteria for CRPC according to the international guidelines [17], (d) at least 3 cycles of ^223^Ra-therapy administered, (e) at least six symptomatic bone metastases and no known visceral metastases, except for malignant lymphadenopathy with less than 3 cm in the short-axis diameter, (f) an Eastern Cooperative Oncology Group (ECOG) performance status (PS) score of 0–1 and adequate hematological, liver and renal function, (g) complete imaging and laboratory follow-up; in particular, clinical and biochemical parameters including PSA, ALP, NLR had to be available for patients’ inclusion.

All the subjects included in the analysis were submitted to therapy with ^223^Ra-dichloride (Xofigo^®^, Bayer AG, Germany), encompassing six intravenous injections of the radiopharmaceutical at a standard dose of 55 kBq/kg at four-week intervals until unacceptable toxicity or worsening of the overall performance status. The use of androgen deprivation therapy (ADT) continued during radionuclide treatment. Concomitant treatment with abiraterone and enzalutamide was not allowed. Patients also received the best standard of care, including glucocorticoids and analgesics.

### 2.2. PET/CT Imaging Procedure

All patients were submitted to PET/CT examination within 3 weeks before the start of ^223^Ra-therapy. All subjects fasted at least 4 h before tracer administration and underwent PET-CT scan 60 min after the intravenous administration of 3.7 KBq/kg of ^18^F-choline (18fluoromethylcholine/Cholscan^®^, Advanced Accelerator Applications, Venafro, Italy).

The PET/CT device consisted of a Discovery ST (GE, Milwaukee, WI, USA) with bismuth germanate crystal units arranged to form 24 rings combined with a 16-slice Light Speed Plus CT scanner. The average full width at half maximum (FWHM) axial resolution of PET (full width at half maximum) is 5.2 mm and system sensitivity 9.3 cps/KBq for 3D acquisition mode. Scanning was performed in 3D modality, with an acquisition time of 3 min per bed/position. Images were reconstructed using an ordered subset expectation maximization iterative algorithm (OSEM-SV, VUEPoint HD, GE, 2 iterations, 15 subsets). The CT was performed immediately before PET in the identical axial field of view using a standardized protocol consisting of automatic tube current modulation with auto mA, tube rotation time of 0.5 s/rotation, slice thickness of 3.75 mm. The CT data were resized from 512 × 512 to a 256 × 256 matrix to match the PET data. The data were transmitted to a nuclear medicine database, fused and displayed using dedicated software (PET VCAR, Advantage Workstation 4.7; GE Healthcare, Milwaukee, WI, USA).

### 2.3. PET-Derived Parameters Calculation

Images were interpreted by 2 experienced nuclear medicine physicians (L.F. and O.B.). Focal or diffuse ^18^F-choline uptake above the background, excluding normal physiological uptake was considered as a positive lesion. The following parameters were carried out from baseline PET/CT analysis: number of lesions detected on PET/CT scan, SUVmax, SUVmean, SULpeak, MATV and TLA. MATV was defined on the pre-treatment PET scan using a dedicated software (PET VCAR, Advantage Workstation 4.7; GE Healthcare, Milwaukee, WI, USA). Every lesion was segmented with a threshold of 42% of the maximum SUV value within the lesion’s bounding box. TLA was calculated as the product of MATV × SUVmean. All the lesions were considered and the TLA and MATV calculated were the sum of the TLA and MATV of all the metastatic lesions.

### 2.4. PET Response Assessment and Clinical Follow-Up

Metabolic response to ^223^Ra-therapy was assessed by ^18^F-choline PET/CT scan within 1 month after the end of treatment. Change in TLA (i.e., ΔTLA) between pre-treatment and post-treatment PET scans was calculated according to the following formula: [(pre-treatment TLA-post-treatment TLA)/pre-treatment TLA] × 100. The metabolic response was defined as a 50% or greater reduction in TLA between baseline and post-treatment PET scans. All patients showing a reduction in TLA inferior to the 50% threshold or an increased TLA were considered as non-responders.

All the subjects were subsequently submitted to monthly follow-up through clinical examination and laboratory tests and imaging follow-up by ceCT, bone scintigraphy and/or ^18^F-choline PET/CT examination every 6 months. All patients were monitored until death. A biochemical (ALP and PSA) response was defined as a reduction of ≥30% from the baseline values to the end of ^223^Ra-therapy [18].

### 2.5. Statistical Analysis

The study’s primary endpoint was to assess the role of ^18^F-choline PET-derived parameters to predict survival after ^223^Ra-therapy. Overall survival was calculated from the patients’ enrollment for ^223^Ra-therapy. Statistical analysis was performed using dedicated software (MedCalc 11.3.8.0; MedCalc Software, Mariakerke, Belgium). OS was calculated by the Kaplan–Meier method, measured from the date of the first administration of ^223^Ra-therapy to the death of the patients. Regression analysis and Pearson’s coefficient were used to calculate the correlation between clinical (i.e., PSA, NLR) and PET-derived parameters, Cox proportional hazard regression analysis was applied to identify prognostic factors on survival. Significance was established at *p* < 0.05.

## 3. Results

The interrogation of our database identified 20 mCRPC patients fulfilling inclusion criteria. All the included subjects were submitted to ^223^Ra-therapy between January 2017 and September 2019. Of the enrolled patients, five had been previously treated with chemoterapy, 12 had been previously submitted to anti-androgen therapy with abiraterone acetate and six had been treated with enzalutamide.

The median interval between PET/CT with ^18^F-choline and the start of ^223^Ra-therapy was 17 days.

Clinical-demographic characteristics of patients and the values of clinical and PET-derived quantitative variables are summarized in Table 1.

Eleven patients (55%) completed all the six scheduled cycles of ^223^Ra-therapy (mean administered cycles 4.8 ± 1.3), the remaining nine discontinued radionuclide treatment due to evidence of progressive disease (*n* = 6, i.e., 30%) or hematological toxicity (*n* = 3, i.e., 15%). All the subjects underwent a follow-up PET/CT with ^18^F-choline for response assessment within 4 weeks after the end of ^223^Ra-therapy. Patients were subsequently submitted to monthly hematological/biochemical follow-up and monitoring by imaging every 6 months, or before in case of changes in patients’ symptoms or parameters, with ^18^F-choline PET/CT (*n* = 4), contrast enhanced computed tomography (*n* = 2) or bone scintigraphy (*n*= 5). In such cases, the choice of the most appropriate diagnostic approach was determined by the referring clinician on the basis of the specific clinical issue. During follow-up, all the patients deceased.

### 3.1. Baseline Clinical and PET-Derived Parameters

All the patients presented ^18^F-choline-avid lesions at baseline PET/CT scan. In particular, 18 patients (i.e., 90%) showed exclusive metastatic skeletal localizations, while two (i.e., 10%) had bone lesions and also presented mild tracer uptake in abdominal-pelvic lymph nodes with maximum diameter <3 cm.

As far as it concerns the number of lesions detected on baseline PET/CT scan, 15 subjects had less than 20 localizations, while five presented more than 20 metastases. As regards PET-derived parameters, median SUVmax resulted in 13.8, median SUVmean was 7, median SULpeak was 8.4, median MATV was 48.2 cc, and median TLA resulted in 351.5 g.

**Figure 1:**
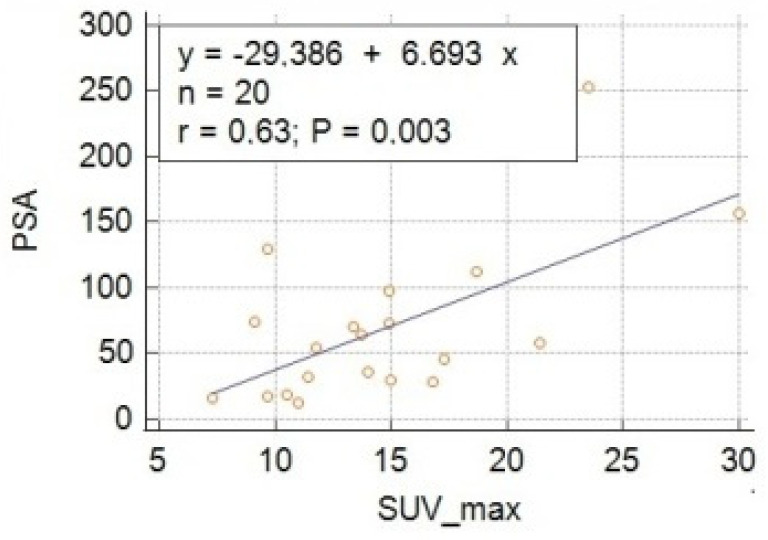
Regression analysis showing the correlation between baseline levels of PSA and maximum standardized uptake value (SUVmax).

From a clinical/biochemical point of view, the median PSA resulted in 50.2 ng/mL and median NLR was 2.9. On regression analysis, a significant positive correlation (i.e., *p* < 0.05) was found between all the PET-derived parameters and PSA levels, with SUVmax being the variable with the strongest correlation (i.e., *r* = 0.63, *p* = 0.003), as shown in Table 2 and Figure 1. No significant correlations were found between PET-derived parameters and NLR.

### 3.2. Predictive Value of Clinical and PET-Derived Parameters on Survival after ^223^Ra-Therapy

**Figure 2:**
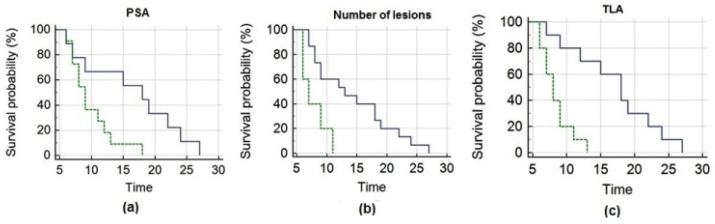
Kaplan–Meier analysis depicts overall survival after ^223^Ra-therapy as a function of pre-treatment PSA (**a**), number of lesions (**b**) and TLA (**c**), respectively. Panel (**a**) shows that patients with PSA ≤ 50.2 ng/mL (blue line) had significantly longer survival than those with PSA > 50.2 ng/mL (green line). Panel (**b**) depicts that subjects with a number of lesions ≤ 20 (blue line) presented significantly longer survival than those presenting > 20 lesions (green line). Panel (**c**) demonstrates that subjects with TLA ≤ 351.5 g (blue line) survived significantly longer than those with TLA > 351.5 g (green line).

The mean OS of the entire cohort of patients was 12.7 ± 1.4 months (median OS = 9 months, 95% CI 7–27). PSA, the number of lesions and TLA significantly correlated with OS (Figure 2). To perform Kaplan–Meier analysis, PET-derived parameters (i.e., SUVmax, SUVmean, SULpeak, MATV, TLA), PSA and NLR were dichotomized at their median value; the other analyzed parameters were dichotomized as shown in Table 3.

Parameters presenting *p* < 0.05 at Kaplan–Meier (i.e., number of lesions, PSA, TLA) were further submitted to multivariate analysis (Cox regression, stepwise): TLA remained the only significant predictor of survival (*p* = 0.003; hazard ratio = 7.6, 95% confidence interval = 1.9–29.5 months).

### 3.3. ^18^F-Choline PET/CT for Response Assessment to ^223^Ra-Therapy

A metabolic response (i.e., ΔTLA > −50%) was detected at post treatment ^18^F-choline PET/CT in seven patients (35%), while the remaining 13 (65%) were non-responders. In the sub-group of non-responders, 10 presented progression at skeletal level, while the remaining three had progressive disease both at the skeletal and visceral level (two with lymph node metastases and one with lymph node and hepatic metastases). In two out of the three patients with visceral involvement a correlative imaging with contrast enhanced CT was performed in order to better define the site and the number of pathological lymph nodes.

All the seven responders continued ADT until evidence of progressive disease, as a result of which they were submitted to chemotherapy (*n* = 6) or therapy with enzalutamide (*n* = 1), while among non-responders, nine were submitted to chemotherapy and the remaining four underwent palliative and supportive care.

All the patients showing metabolic response also presented biochemical response, while none of the non-metabolic responders presented a significant reduction in PSA/ALP value with respect to the baseline levels. In the responders’ cohort, the mean ΔTLA was −72% ± 14%, the mean ΔPSA was −76% ± 10%; on regression analysis, a moderately strong non-significant correlation (r = 0.53, *p* = 0.2) was registered between the two aforementioned parameters (i.e., ΔTLA and ΔPSA).

**Figure 3:**
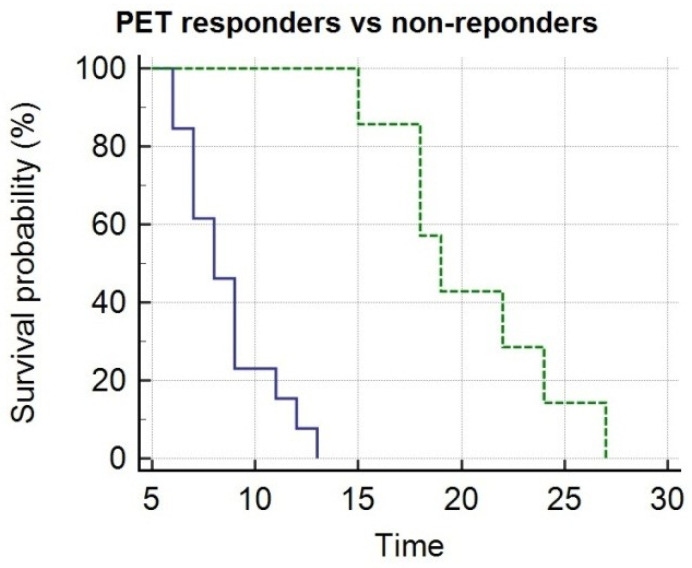
Kaplan–Meier analysis depicts overall survival after ^223^Ra-therapy as function of metabolic response assessed at post treatment PET/CT with ^18^F-choline. PET responders (green line) presented significantly longer survival as compared with non-responders (blue line).

**Figure 4:**
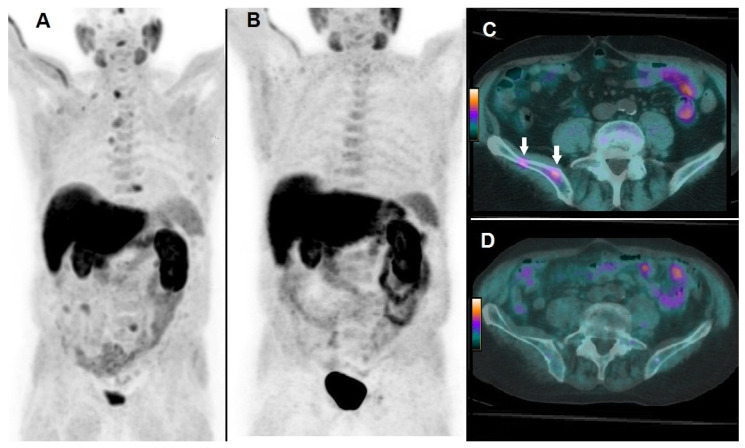
A 74-year-old patient affected by mCRPC, previously treated with androgen deprivation therapy, subsequently submitted to ^223^Ra-therapy. At baseline, ^18^F-choline PET/CT demonstrated multiple sites of tracer uptake in bones, as evident in Whole Body (**A**) and the following parameters were registered: PSA = 17 ng/mL; number of lesions = 18; TLA = 235.3 g. ^18^F-choline PET/CT acquired after 6 cycles of ^223^Ra-therapy demonstrated complete metabolic response as depicted by Whole Body (**B**). ^18^F-choline PET/CT fused axial images of the pelvis acquired at baseline showed hypermetabolic lesions in the right iliac bone (**C**, white arrows), while post-treatment axial slices did not show any pathological sites of tracer uptake (**D**). Post-treatment PSA was 3 ng/mL, OS resulted in 27 months.

**Figure 5:**
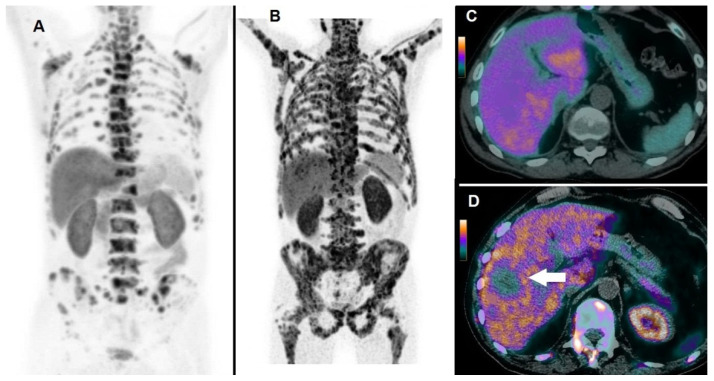
A 66-year-old patient affected by mCRPC, previously treated with abiraterone acetate and docetaxel, then submitted to ^223^Ra-therapy. ^18^F-choline PET/CT acquired at baseline demonstrated multiple tracer uptake sites in bones, as evident in Whole Body (**A**); baseline pre-treatment parameters were as follows: PSA = 157 ng/mL; the number of lesions > 20; TLA = 3969.9 g. After 3 cycles, ^223^Ra-therapy was discontinued due to deterioration of patient’s performance status and increased PSA (i.e., 289 ng/mL). ^18^F-choline PET/CT acquired after 3 cycles of ^223^Ra-therapy demonstrated an impressive progression of skeletal disease, as shown by Whole Body (**B**). ^18^F-choline PET/CT fused images of the liver acquired at baseline did not show abnormal tracer uptake (**C**), while post-treatment axial slices demonstrated the appearance of a partially necrotic metastasis in the VIII/VII hepatic segment (**D**, white arrow). The patient’s OS resulted in 7 months.

By Kaplan–Meier analysis, responders presented a significantly longer survival than non-responders (i.e., median OS = 19 months, 95% CI 15–27, vs. 8 months, 95% CI 7–13, *p* < 0.05), as shown by Figure 3. Emblematic examples of ^18^F-choline PET/CT findings in a patient with more favorable prognostic factors and clinical outcome are depicted in Figure 4, while Figure 5 describes a subject with poor prognostic factors and outcome.

### 3.4. Toxicities

Among the enrolled subjects, three cases (15%) of hematological toxicity were registered, one (5%) of whom was a grade 2 Hb toxicity not needing transfusion, since it spontaneously regressed after ^223^Ra-therapy discontinuation; the remaining two cases (10%) presented, respectively, grade 3 Hb toxicity and pancytopenia (grade 3 Hb and PLT toxicity and grade 2 lymphocyte toxicity) and were treated with transfusions.

## 4. Discussion

A growing amount of scientific data suggests that the clinical efficacy of ^223^Ra-therapy in real-life practice is strictly dependent on pre-treatment patients’ prognostic stratification [12,19]. This issue calls for an unmet need for clinical and imaging biomarkers suitable for identifying patients who can benefit from such a therapeutic regimen.

Although PET/CT scan with ^18^F-choline is routinely utilized for the detection of prostate cancer recurrence, its use for the prognostication of patients submitted to ^223^Ra-therapy is yet to be defined. To the best of our knowledge, this is the first study analyzing the prognostic role of ^18^F-choline PET-derived parameters on patients’ survival after ^223^Ra-therapy, showing that baseline overall burden of disease, as expressed by TLA, has an independent predictive impact on multivariate Cox analysis.

In a prospective study by Garcia et al. [20], 40 patients affected by mCRPC were submitted to bone scintigraphy and ^18^F-choline PET/CT prior to ^223^Ra-therapy. The following parameters were calculated on baseline PET/CT scan: number of lesions, SUVmax and average SUVmax. All the aforementioned quantitative data were significantly associated with survival after radionuclide therapy. Our results confirm disease extent, expressed by the number of lesions detected on PET/CT, as a relevant prognostic factor on Kaplan–Meier analysis; on the contrary, in our cohort SUVmax was not significantly correlated with OS. This discrepancy might be explained by the fact that SUVmax reflects only the lesion portion with the highest metabolic activity and does not provide information on volumetric tumor features. As a matter of fact, Garcia and collaborators in their analysis did not take into account MATV and TLA, which combine volumetric and functional information.

Caroli et al. retrospectively assessed the predictive value of ^18^F-choline PET-derived parameters in 94 mCRPC patients submitted to therapy with abiraterone acetate or enzalutamide: among the analyzed PET-derived parameters (SUVmax, SUVmean, TLA, MATV), the authors found that the sum of MATV (SMATV) and TLA (STLA) were correlated with survival at univariate analysis, but STLA (median value 495.070 g) remained the only predictor of survival on multivariate analysis [13].

Quaquarini and coworkers investigated the prognostic role of PET/CT with ^18^F-choline in 29 mCRPC patients submitted to chemotherapy with docetaxel, through the calculation of several PET-derived parameters: both SMATV (i.e., median 27.1 cc) and STLA (i.e., median 253.5 g) correlated with progression-free survival [16]. It has to be pointed out that, although the authors used a different terminology in the previously cited studies [13,16], both SMATV and STLA correspond to the parameters calculated in our study (i.e., MATV and TLA, respectively), reflecting the overall burden of metabolically active disease. Our results support the findings reported in the aforementioned papers, which indicate TLA as a relevant prognostic factor in mCRPC submitted to systemic therapy.

Further consideration should be made regarding the different approaches applied for the calculation of PET-derived volumetric parameters. Racaru et al. assessed the value of ^18^F-choline PET/CT for predicting toxicity associated with ^223^Ra-therapy [14]: in a group of 15 patients, MATV and TLA were shown to be capable of predicting Hb and PLT toxicity and were therefore suggested as potential tools for patients’ selection before treatment. In the previously cited paper, MATV was carried out through an open-source software, including voxels with an SUV of at least 3 and corresponding to a CT value more than – 1000 Hounsfield. In contrast to Racaru’s group, we used a segmentation method based on a fixed threshold of 42% of the SUVmax, substantially similar to that applied by Caroli et al. [13]. It is still debated which is the best approach for volumetric segmentation in order to obtain the most robust and reproducible results; further studies are needed to better define this technical issue.

Lastly, although the primary endpoint of our research was focused on survival, in our cohort of mCRPC patients, PET/CT with ^18^F-choline was shown to be a reliable approach for assessing response to ^223^Ra-therapy. In particular, as previously demonstrated by a preliminary report from our group [15], a reduction of at least 50% of the overall metabolically active tumor burden (i.e., TLA) after ^223^Ra-therapy correlated with a significantly longer survival. Of note, we found that all PET-responders presented a significant reduction in PSA/ ALP levels with respect to the baseline values, with a good correlation, although not statistically significant, between ΔTLA and ΔPSA. It has to be underlined that several reports indicate that PSA dosage might not represent a reliable approach for monitoring the response to treatment, since it may present a transitory increase after the first cycle of ^223^Ra-therapy, in relation to the so-called “PSA flare” due to the release of PSA from tumor cell lysis [21]. The correlation between ΔTLA and ΔPSA found in our cohort might be explained by the inclusion criteria we applied for the enrollment, since only patients who had completed three cycles of ^223^Ra-therapy were considered, thus minimizing the interference of “PSA flare” occurring in the first month of treatment.

In this regard, the bone-seeking radiopharmaceutical ^18^F-fluoride has been applied by Kairemo et al. [22] to assess tumor response in 10 subjects treated with ^223^Ra-therapy. The authors performed ^18^F-fluoride PET/CT at baseline, after the 1st and the 6th cycle and applied a modified version of PET response evaluation criteria (PERCIST). While PET/CT after the 1st cycle did not provide additional information, PET evaluation after the 6th cycle demonstrated metabolic response in all patients, with a correlation with PSA trend in 9/10 cases.

The main drawback of PET/CT with ^18^F-fluoride is represented by its capability to monitor only the metabolic response of bone metastases to ^223^Ra-therapy, while it is unable to detect eventual progression at the visceral level. In our cohort of patients, for example, ^18^F-choline PET/CT was shown to be useful in identifying the appearance of visceral metastases in 3 out of the 13 non-responder patients (i.e., 23%).

Our study presents some limitations, such as the small cohort of patients and its retrospective nature. Furthermore, clinical parameters other than those included in this retrospective analysis (such as LDH) might impact patients’ prognostic stratification. Further studies with larger cohorts are needed to better define the role of ^18^F-choline PET/CT for selection before ^223^Ra-therapy.

## 5. Conclusions

^18^F-choline PET/CT may represent a useful tool for patients’ prognostic stratification before ^223^Ra-therapy and for the assessment of response to treatment. In particular, PET-derived parameter TLA is an independent predictor of survival in multivariate analysis.

## Figures and Tables

**Table 1:** Patients’ clinical-demographic features and results of quantitative parameters.

**Age (years)**		-
Median	75	
mean ± SD	74.7 ± 3.7	
**Baseline ECOG PS**	***n***	**%**
0	16	80%
1	4	20%
**Gleason Score**	***n***	**%**
8–9	14	70%
6–7	6	30%
**Previous Therapies**	***n***	**%**
Chemotherapy	5	25%
Enzalutamide	6	30%
Abiraterone acetate	12	60%
Zolendronate	4	20%
Denosumab	2	10%
**Baseline levels ALP (U/L)**	***n***	**%**
<150	18	90%
>150	2	10%
**Baseline levels of PSA (ng/mL)**	mean ± sd	median
	69.2 ± 62.5	50.2
**Baseline neutrophil to lymphocyte ratio**	mean ± sd	median
	3.3 ± 1.1	2.9
**Number of lesions on baseline PET/CT**		
<20	15	75%
>20	5	25%
**Baseline PET-derived parameters**	**mean ± sd**	**median**
SUVmax	14.7 ± 5.4	13.8
SUVmean	7.2 + 2.2	7
SULpeak	10.5 ± 8.5	8.4
MATV (cc)	80.2 ± 83.7	48.2
TLA (g)	583 ± 836.4	351.5

Abbreviations: ECOG PS—Eastern Cooperative Oncology Group Performance Status; ALP—alkaline phosphatase, PSA—prostate specific antigen; SUVmax—maximum standardized uptake value; SUVmean—mean standardized uptake value; SULpeak—lean body mass corrected SUV peak; MATV—whole body metabolically active tumor volume; TLA—total lesion activity.

**Table 2:** PET-derived parameters and PSA levels at baseline: regression analysis.

PET Parameters	r	*p* *
SUVmax	0.63	0.003
SUVmean	0.56	0.01
SULpeak	0.51	0.02
MATV	0.45	0.04
TLA	0.51	0.02

* Significant at *p* < 0.05.

**Table 3:** Results of Kaplan–Meier analysis of clinical and PET-derived parameters.

	No. of Patients	Overall Survival (months), Mean (95% CI)	*p*
Gleason score			
8–9	14	11.1 (7.5–14.7)	0.34
6–7	6	16.5 (13.4–19.5)	
Previous CHT			
No	16	13.8 (10.5–17.1)	0.09
Yes	4	8.5 (5.9–11)	
Hb levels			
≤12 g/L	6	9.5 (7.4–11.5)	0.1
>12 g/L	14	14.1 (10.3–17.9)	
Number of lesions			
≤20	15	14.4 (11–17.7)	0.01 *
>20	5	7.8 (5.9–9.7	
ALP levels			
≤150 U/L	18	13.3 (10.3–16.3)	0.1
>150 U/L	2	7.5 (4.5–10.4)	
PSA levels			
≤50.2 ng/mL	9	16.3 (11.3–21.3)	0.04 *
>50.2 ng/mL	11	9.8 (7.7–11.8)	
SUVmax			
≤13.8	10	14.3 (10.3–18.2)	0.3
>13.8	10	11.2 (7.1–15.2)	
SUVmean			
≤7	10	14.3 (10.3–18.2)	0.3
>7	10	11.2 (7.1–15.2)	
SULpeak			
≤8.4	12	13.9 (10.1–17.6)	0.3
>8.4	8	11 (6.6–15.3)	
MATV			
≤48.2 (cc)	9	16 (12.2–19.7)	0.1
>48.2 (cc)	11	10 (6.5–13.6)	
TLA			
≤351.5 (g)	10	17.1 (13.1–21)	0.003 *
>351.5 (g)	10	8.4 (7–9.7)	

* *p* was significant at statistical analysis.

## Abbreviations

mCRPC	Metastatic castration resistan prostate cancer
PET/CT	Positron emission computed tomography
TLA	Total lesion glycolysis
MATV	Metabolically active tumor volume
